# Activation of TRPA1 by membrane permeable local anesthetics

**DOI:** 10.1186/1744-8069-7-62

**Published:** 2011-08-23

**Authors:** Andreas Leffler, Anja Lattrell, Sergej Kronewald, Florian Niedermirtl, Carla Nau

**Affiliations:** 1 Department of Anesthesiology, Friedrich-Alexander-University Erlangen-Nuremberg, Krankenhausstrasse 12, 91054 Erlangen, Germany; 2 Department of Anesthesiology and Intensive Care, Hannover Medical University, Carl-Neuberg-Str. 1, 30625 Hannover, Germany

## Abstract

**Background:**

Low concentrations of local anesthetics (LAs) suppress cellular excitability by inhibiting voltage-gated Na^+ ^channels. In contrast, LAs at high concentrations can be excitatory and neurotoxic. We recently demonstrated that LA-evoked activation of sensory neurons is mediated by the capsaicin receptor TRPV1, and, to a lesser extent by the irritant receptor TRPA1. LA-induced activation and sensitization of TRPV1 involves a domain that is similar, but not identical to the vanilloid-binding domain. Additionally, activation of TRPV1 by LAs involves PLC and PI(4,5)P_2_-signalling. In the present study we aimed to characterize essential structural determinants for LA-evoked activation of TRPA1.

**Results:**

Recombinant rodent and human TRPA1 were expressed in HEK293t cells and investigated by means of whole-cell patch clamp recordings. The LA lidocaine activates TRPA1 in a concentration-dependent manner. The membrane impermeable lidocaine-derivative QX-314 is inactive when applied extracellularly. Lidocaine-activated TRPA1-currents are blocked by the TRPA1-antagonist HC-030031. Lidocaine is also an inhibitor of TRPA1, an effect that is more obvious in rodent than in human TRPA1. This species-specific difference is linked to the pore region (transmembrane domain 5 and 6) as described for activation of TRPA1 by menthol. Unlike menthol-sensitivity however, lidocaine-sensitivity is not similarly determined by serine- and threonine-residues within TM5. Instead, intracellular cysteine residues known to be covalently bound by reactive TRPA1-agonists seem to mediate activation of TRPA1 by LAs.

**Conclusions:**

The structural determinants involved in activation of TRPA1 by LAs are disparate from those involved in activation by menthol or those involved in activation of TRPV1 by LAs.

## Background

LAs are well established in clinical practice and their use is accompanied by a low prevalence of severe side effects. However, it is well documented that intrathecal and perineural application of highly concentrated LAs can be neurotoxic and induce sequelae such as transient neurological symptoms and the cauda equina syndrome [1-4]. Several cellular mechanisms have been suggested to mediate LA-induced neurotoxicity. The most recent reports suggest that LAs can induce both necrosis and apoptosis [5-9]. In dorsal root ganglion (DRG) neurons, lidocaine-induced cell death was also reported to be associated with a Ca^2+^-influx and a depolarization [10]. Moreover, recent studies propose that LAs can enhance a release of neuropeptides when applied into surgical wounds and induce a release of glutamate from central sensory terminals when applied intrathecally [11,12]. Accordingly, the local injection of most clinically established formulations of local anesthetics is accompanied by a brief, but discomforting burning sensation [13,14]. Our laboratory recently reported that LAs directly activate the capsaicin receptor TRPV1 and evoke a TRPV1-dependent release of neuropeptides from mouse skin and from isolated peripheral nerves [15]. TRPV1 is selectively expressed in nociceptive sensory neurons where it acts as a polymodal membrane receptor for noxious heat and various chemical insults [16,17]. The pore of TRPV1 displays an unselective permeability for mono- and divalent cations, but is also permeable for large molecules such as the lidocaine derivative QX-314 [18,19]. Due to this property, TRPV1 can mediate neurotoxicity by mechanisms involving an intracellular Ca^2+^-overload and a consecutive apoptosis or necrosis [20,21]. Thus TRPV1 is a candidate mediator of LA-evoked neurotoxicity, injection pain and release of neuropeptides [15]. However, LAs also evoke a TRPV1-independent activation of a subpopulation of sensory neurons that is due to activation of the irritant receptor TRPA1 [15]. Furthermore, Piao and colleagues suggested that LA-induced release of glutamate from central sensory terminals is mediated by TRPA1 rather than by TRPV1 [11]. In this study we therefore aimed to validate and further characterize the effects of LAs on TRPA1 by means of conventional whole-cell patch clamp recordings on recombinant rodent and human TRPA1.

## Results

**Figure 1:**
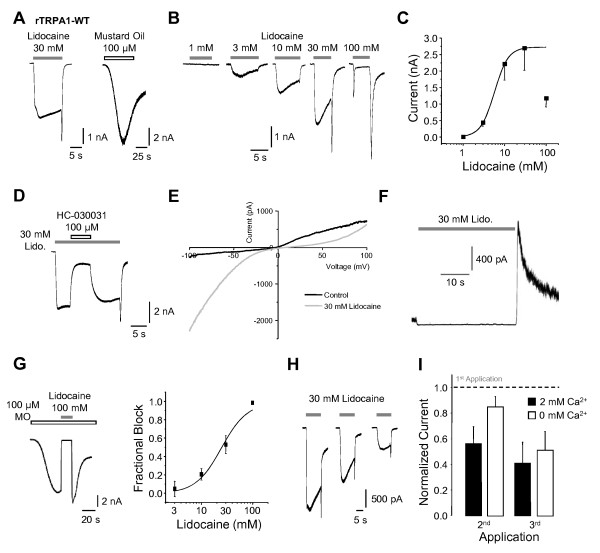
**Lidocaine activates and blocks rat TRPA1**. **A**. Representative inward currents in HEK293t cells expressing rTRPA1 activated by 30 mM lidocaine and 100 μM mustard oil. Cells were held at -60 mV. **B- C**. Concentration dependent activation of rTRPA1 by lidocaine. Only one concentration was applied on each cell. The Hill-equation was applied to calculate the EC_50 _value. **D**. A typical example of a lidocaine-evoked current blocked by the selective TRPA1-anatagonist HC-030331 (100 μM). **E**. Ramp currents of rTRPA1 in control solution and during application of 30 mM lidocaine. Cells were held at -60 mV and currents were measured during 500 ms long voltage-ramps from -100 to +100 mV. Note the lack of an outward rectification of the lidocaine-evoked current **F**. Representative effects on rTRPA1 30 mM lidocaine in cells held at +60 mV..**G**. Typical MO-evoked inward current blocked by 100 mM lidocaine. Increasing concentrations of lidocaine were co-applied with MO during the steady-state phase of MO-evoked currents. Experiments were performed in a Ca^2+^-free extracellular solution to minimize desensitization. The fractional block was plotted against the lidocaine concentration. The line represents the fit of the data to the Hill equation. **H**. Representative currents of TRPA1-currents activated by three consecutive applications of 30 mM lidocaine in standard extracellular solution containing 2 mM Ca^2+^. Lidocaine was applied in intervals of 2 min. **I**. Mean current amplitudes of rTRPA1 currents evoked by repeated applications of lidocaine in standard extracellular solution containing 2 mM Ca^2+ ^or in a Ca^2+^-free solution. Current amplitudes are normalized to the value obtained with the first application of lidocaine (dotted line).

HEK293t cells expressing wildtype rat TRPA1 (rTRPA1) were examined by standard whole cell voltage-clamp recordings (V_h _-60 mV). As demonstrated in Figure 1A, the LA lidocaine (30 mM) activates inward currents in these cells being also activated by the TRPA1-agonist mustard oil (MO, 100 μM). This lidocaine-evoked activation of rTRPA1 is concentration-dependent as demonstrated by increasing peak amplitudes of currents evoked by 3 mM (432 ± 104 pA, n = 6), 10 mM (2211 ± 483 pA, n = 8) and 30 mM (2690 ± 668 pA, n = 8). At 100 mM however, lidocaine evokes rapidly inactivating currents which are generally smaller (1169 ± 257 pA, n = 8) than those activated by 30 mM (Figure 1B). The calculated EC_50_-value of lidocaine-evoked activation of rTRPA1 is 5.7 ± 0.2 mM (Figure 1C, Hill-equation, n = 22). Importantly, non-transfected cells do not produce any lidocaine-evoked inward currents in concentrations up to 100 mM. Moreover, hyperosmolaric solutions containing glucose up to a concentration of 130 mM did not evoke any activation of rTRPA1 (n = 5, data not shown). The TRPA1-inhibitor HC-030031 (100 μM) blocks lidocaine-induced rTRPA1-currents (by 64 ± 5%, n = 5, Figure 1D). Furthermore, rTRPA1 is also activated by the local anesthetics mepivacaine and procaine (30 mM in each case, data not shown). Notably, the inward currents evoked by 30 and 100 mM lidocaine display large resurging currents following washout of lidocaine (Figure 1B). rTRPA1 also exhibits a less pronounced outward-rectification in presence of 30 mM lidocaine as compared to control solution during 500 ms-long voltage-ramps from -100 to +100 mV (Figure 1E). Furthermore, 30 mM lidocaine failed to evoke outward currents in cells held at +60 mV, however produced large resurging outward current following washout (Figure 1F). These data are reminiscent of a bimodal effect of lidocaine on TRPA1, as was also described for TRPV1 [15]. As demonstrated in Figure 1G, lidocaine blocked TRPA1-mediated currents activated by mustard oil (100 μM). This block displays a rapid onset, is reversible upon washout of lidocaine and is concentration dependent (Figure 1G, IC_50_-value 25 ± 3 mM, n = 4- 5 for each concentration). Other studies suggest that the dilated pore state of TRPA1 is Ca^2+^-dependent. Moreover, it is the dilated pore that enables more efficient open channel block [22]. We found that block of MO-evoked currents by 30 mM lidocaine is comparable in the presence or absence of extracellular Ca^2+^. (data now shown). Thus, a dilated pore is not a prerequisite for lidocaine-induced block of mTRPA1.

We also observed a pronounced tachyphylaxis and desensitization of lidocaine-evoked activation of rTRPA1 upon repetitive applications (Figure 1H). While a similar tachyphylaxis and desensitization of LA-evoked TRPV1-currents were found to be strongly enhanced in the absence of extracellular calcium [15], the removal of extracellular calcium rather seems to reduce this process on rTRPA1 (Figure 1I, p < 0.05 for 2^nd ^application, unpaired t-test).

**Figure 2:**
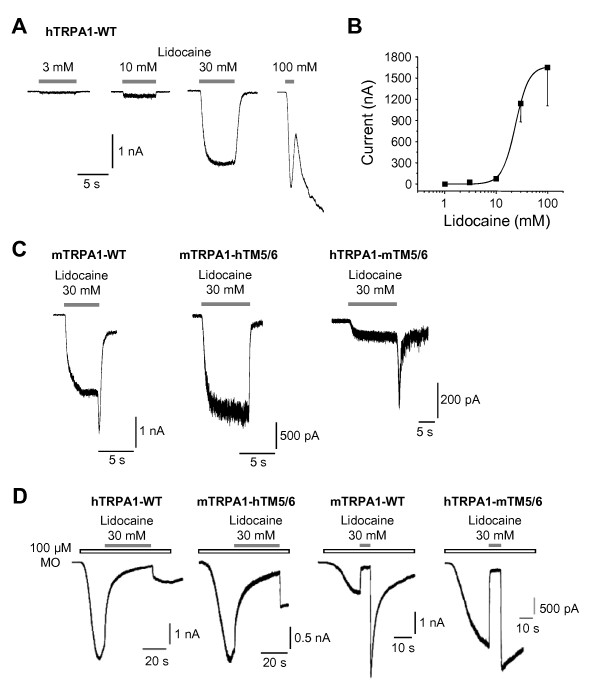
**Lidocaine activates human TRPA1**. **A**. Representative inward currents in HEK293t cells expressing hTRPA1 activated by 3, 10, 30 and 100 mM lidocaine. Cells were held at -60 mV and only one concentration was applied on each cell. **B**. Concentration-dependent activation of hTRPV1 by lidocaine. The Hill-equation was applied to calculate the EC_50 _value. Transmembrane domain 5 is a determinant for species different activation of TRPA1 by lidocaine **C**. Representative inward currents evoked by 30 mM lidocaine on mTRPA1-WT, and the chimeras mTRPA1-hTM5/6 and hTRPA1-mTM5/6. Note the lack of a resurging current for mTRPA1-hTM5/6. **D**. Typical experiments for lidocaine-induced block of MO-evoked inward currents on hTRPA1-WT, mTRPA1-hTM5/6, mTRPA1-WT and hTRPA1-mTM5/6. Lidocaine was co-applied with MO during the steady-state phase of MO-evoked currents and experiments were performed in a Ca^2+^-free extracellular solution to minimize desensitization. Note the slow onset of lidocaine-induced block for hTRPA1-WT and mTRPA1-hTM5/6 as compared to mTRPA1-WT and hTRPA1-mTM5/6. Cells were held at -60 mV.

We next examined the effects of lidocaine on human TRPA1 (hTRPA1). Similar to rTRPA1, hTRPA1 is activated by lidocaine ≥ 3 mM (3 mM 36 ± 8 pA, n = 11; 10 mM 86 ± 12 pA; n = 9, 30 mM 1147 ± 266 pA, n = 18; 100 mM 1652 ± 541 pA, n = 10) with a calculated EC_50_-value of 24.0 ± 0.6 mM (Figure 2A and 2B, Hill-equation). Notably, the resurging current following washout of lidocaine observed for rTRPA1 is completely missing for hTRPA1 (Figure 2A). A similar species-specific effect on TRPA1 was recently reported for menthol, i.e. menthol activates and blocks mouse TRPA1 (mTRPA1) while hTRPA1 is only activated [23]. This difference between mTRPA1 and hTRPA1 was linked to transmembrane domain 5 (TM5) which was also suggested to be a crucial interaction site for menthol-evoked activation of TRPA1 [23]. We therefore asked if lidocaine and menthol employ common mechanisms to activate TRPA1. For this reason, we explored mutant constructs of mouse and human TRPA1. Wildtype mTRPA1, similar to rTRPA1, generates lidocaine-evoked (30 mM) responses with resurging currents (1889 ± 742 pA, n = 9) (Figure 2C). The chimera mTRPA1-hTM5/6, in which the region TM5 through TM6 of hTRPA1 was introduced, similar to wildtype hTRPA1 generates lidocaine-evoked currents without any resurging currents (1095 ± 292 pA, n = 7). Accordingly, the reverse swap of mTM5/6 into hTRPA1 results in small lidocaine-evoked currents which are followed by prominent resurging currents (54 ± 11 pA, n = 6) (Figure 2C). Consistent with the hypothesis that the resurging current is a result of a bimodal effect of lidocaine on TRPA1, MO-activated currents of mTRPA1-WT and hTRPA1-mTM5/6 are blocked by lidocaine (89 ± 4% block, n = 11 and 87 ± 5% block, n = 8, respectively) and show a resurging current following washout of lidocaine. The prominent effect of lidocaine on MO-activated currents of hTRPA1-WT and mTRPA1-hTM5/6 however seems to be an activation, followed by an acute desensitization (Figure 2D and Additional file 1, figure s1). We next examined if residues (S876/T877) within TM5, which were demonstrated to be required for activation by menthol [23], are also required for activation by lidocaine. Surprisingly, the menthol-insensitive mutant mTRPA1-S876V/T877L generates large lidocaine-evoked inward currents with a mean current amplitude (582 ± 151 pA, n = 6, data not shown) not significantly smaller than the mean current amplitude of mTRPA1-WT (p = 0.16, unpaired t-test). Thus, although menthol and lidocaine interact with common sites within TM5, they seem to employ distinct mechanisms to activate TRPA1.

We intended to test whether residues S876/T877 are involved in the LA-evoked block of MO-activated mTRPA1. Surprisingly, we found that 30 mM lidocaine does not block MO-induced mTRPA1-S876V/T877L currents but in contrast evoked an increase in the MO-induced current followed by an acute desensitization (Additional file 2, figure s2). A resurging current following washout of lidocaine however suggests a bimodal effect of lidocaine on mTRPA1-S876V/T877L. These data indeed indicate that residues S876/T877 might be involved in the LA-evoked block of mTRPA1, although other sites might contribute as well. As the effect of lidocaine on mTRPA1-S876V/T877L strongly resembles the effect on hTRPA1, this result underlines the species-specific involvement of transmembrane domains 5 and 6 in gating and blocking mechanisms of TRPA1.

**Figure 3:**
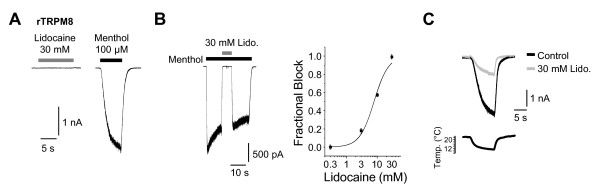
**Lidocaine blocks but does not activate TRPM8**. **A**. Typical recordings on rTRPM8 with a completely lacking response to 30 mM lidocaine but with a large inward current evoked by 100 μM menthol. Cells were held -60 mV **B**. Representative current trace displaying lidocaine-induced block of a menthol-evoked inward current on rTRPM8. Lidocaine was co-applied with menthol during the steady-state phase of menthol-evoked currents. The fractional block was plotted against the lidocaine concentration. The line represents the fit of the data to the Hill equation. **C**. Cold-induced inward currents of rTRPM8 in control solution (black) or in presence of 30 mM lidocaine (grey). The applied solution was cooled from room temperature (~ 24°C) to ~12°C within 10 seconds. Cells were held at -60 mV.

We also asked if lidocaine and menthol exert similar effects on the related menthol-sensitive receptor TRPM8. As is demonstrated in Figure 3A, however, lidocaine does not activate inward currents in HEK293t cells expressing rat TRPM8. However, lidocaine acts as blocker on rTRPM8 activated by menthol (Figure 3B, IC_50_-value 8 ± 1 mM, n = 4- 7 for each concentration) and by cold (Figure 3C, 64 ± 4% block by 30 mM lidocaine, n = 6).

**Figure 4:**
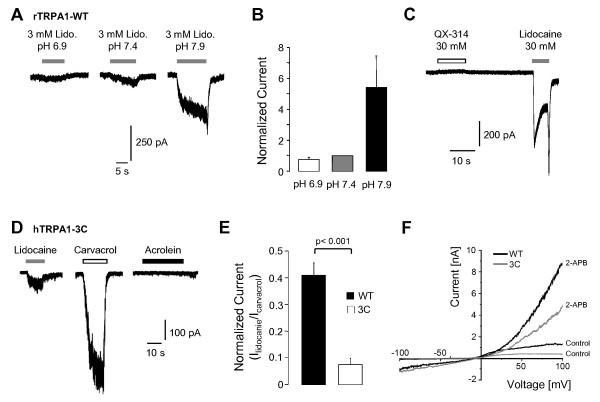
**Intracellular interaction sites of lidocaine on TRPA1**. **A**. Current traces of wildtype rTRPA1 activated by 3 mM lidocaine at pH 6.9, 7.4 or 7.9. Lidocaine-solutions with different pH-values were applied on the same cells in intervals of 1 min. **B**. Relative current amplitudes of rTRPA1 activated with 3 mM at different pH-values. The absolute current amplitudes were normalized to the amplitude determined for pH 7.4. **C**. Typical effect of the impermeable lidocaine-derivative QX-314 on rTRPA1. 30 mM QX-314 did not evoke any current responses in TRPA1-expressing HEK293t cells in which 30 mM lidocaine evoked large inward currents. Cells were held at -60 mV. **D**. Representative experiment performed on the acrolein-insensitive mutant construct hTRPA1-C621S/C641S/C665S (hTRPA1-3C). The cells were treated with 30 mM lidocaine, 250 μM carvacrol and 50 μM acrolein in intervals of 1 min. **E**. Relative current amplitudes of lidocaine-evoked currents on hTRPA1-WT and the hTRPA1-3C construct. Peak current amplitudes determined for lidocaine-evoked currents were normalized with the amplitudes of carvacrol-evoked currents in the same cells. Lidocaine (30 mM) and carvacrol (250 μM) were applied in intervals of 1 min. Cells held at -60 mV. **F**. Ramp currents of hTRPA1-wildtype (WT) and hTRPA1-3C (3C) in control solution and during application of 1 mM 2-APB. Cells were held at -60 mV and currents were measured during 500 ms long voltage-ramps from -100 to +100 mV.

LAs activate TRPV1 via an intracellular pathway and the intracellularly located capsaicin-binding domain seems to be crucially involved in this process [15]. While the capsaicin-binding domain is not conserved in TRPA1, intracellular cysteine residues have been suggested to be important interaction sites for reactive TRPA1-agonists like mustard oil and acrolein [24,25]. In order to examine whether LAs activate TRPA1 via intracellular mechanisms, HEK293t cells expressing rTRPA1 were treated with 3 mM lidocaine at pH 6.9, 7.4 and 7.9 (Figure 4A). Lidocaine is a weak base with a pKa-value of 7.9. While only 25% of the total lidocaine is membrane-permeable at pH 7.4, the membrane-permeable fraction is reduced to 10% at pH 6.9 and increased to 50% at pH 7.9. Compared to currents evoked by lidocaine at pH 7.4, currents are smaller at pH 6.9 (0.8 ± 0.2-fold, n = 5) and significantly larger at pH 7.9 (5 ± 2-fold, n = 5, p < 0.001, paired t-test) (Figure 4B). These data imply that activation of TRPA1 requires membrane permeability of lidocaine. Accordingly, the membrane impermeable lidocaine derivative QX-314 (30 mM) does not activate TRPA1 when applied extracellularly (Figure 4C, n = 5). We now wanted to find out whether LAs indeed interact with the intracellular cysteine residues C621, C641 and C665 of hTRPA1 as was previously described for reactive TRPA1-agonists [25]. The triple mutant hTRPA1-C621S/C641S/C665S (hTRPA1-3C) is acrolein-insensitive but it still activated by carvacrol (250 μM) (Figure 4D). We observed that in all hTRPA1-3C HEK293t-cells generating robust carvacrol-evoked currents (> 50 pA), currents evoked by 30 mM lidocaine were very small (20 ± 8 pA, n = 11) (Figure 4D). As this apparent reduction in lidocaine-sensitivity can be due to a general loss of functionality of hTRPA1-3C, we compared relative peak amplitudes evoked by lidocaine and carvacrol (i.e. I_liodocaine_/I_carvacrol_) in hTRPA1-3C and wildtype hTRPA1. As demonstrated in Figure 4E, the relative lidocaine-sensitivity of hTRPA1-3C (I_lidocaine_/I_carvacrol _= 7.5 ± 2.3%, n = 11) is indeed significantly reduced as compared to the wildtype construct (I_lidocaine_/I_carvacrol _= 40.9 ± 4.7%, n = 10, Figure 4E) (p < 0.001, unpaired t-test). To further test the functionality of hTRPA1-3C to other modes of activation, we also investigate activation by 2-APB (2-Aminoethoxydiphenyl borate) and by voltage. As demonstrated by two representative experiments in Figure 4F, both hTRPA1-wildtype (n = 6) and hTRPA1-3C (n = 6) display robust outward currents when challenged with a 500 ms long voltage ramp from -100 mV to +100 mV. Furthermore, 1 mM 2-APB activated currents with a profound outward rectification on hTRPA1-wildtype and hTRPA1-3C (Figure 4F). We thus can exclude a loss of functionality of the mutant to activation by 2-APB and voltage. These data thus indicate that lidocaine indeed might interact with intracellular cysteines to gate TRPA1, however, the residual lidocaine-sensitivity of hTRPA1-3C suggests that further mechanisms are involved in LA-induced activation.

There is strong evidence suggesting that intracellular Ca^2+^, increased by LA-mediated PLC activation, might be a downstream signalling element of LA-evoked activation of TRPA1 [26,27]. For this reason, we have finally tested the LA-sensitivity in a mutation within the N-terminal EF-hand Ca^2+^-binding domain, hTRPA1-L474A. This mutation is insensitive to Ca^2+^-dependent modulation of agonist-induced responses, is insensitive to icilin and renders TRPA1 insensitive to activation by Ca^2+ ^[28]. We found that hTRPA1-L474A is activated by lidocaine in a concentration-dependent manner (Additional file 3, figure s3). These findings do not suggest that Ca^2+ ^primarily induces activation of TRPA1 by LAs.

## Discussion

TRPV1 and TRPA1 have emerged as two principle molecules for the perception of pungent and irritant substances in nociceptive sensory neurons [29,30]. In the meantime, an impressive number of agonists for each receptor have meanwhile been identified. Along with these discoveries, several distinct gating mechanisms have been revealed for both proteins. We could now demonstrate that local anesthetics directly activate TRPA1 and that they are thus common agonists of TRPA1 and TRPV1 [15].

We previously reported that high concentrations of LAs activate acrolein-sensitive (i.e. TRPA1-expressing) dorsal root ganglion neurons derived from TRPV1-knockout mice [15]. As demonstrated here, LAs indeed activate both rodent and human TRPA1 heterologously expressed in HEK293t cells. Lidocaine-evoked currents were rapidly reversible upon washout of lidocaine, displayed a clear concentration-dependency, were blocked by the TRPA1-agonist HC030031 and were not observed in non-transfected HEK293t cells. Thus there is little doubt that the lidocaine-evoked inward currents are due to a specific activation of TRPA1. Furthermore, lidocaine-evoked TRPA1-currents display properties similar to lidocaine-evoked TRPV1-currents [15]: 1. When heterologously expressed in HEK293t cells, both TRPV1 and TRPA1 are activated by lidocaine > 1 mM and thus seem to have similar lidocaine-sensitivites (IC_50_, _rTRPA1_: 5.7 ± 0.2 mM; IC_50, hTRPA1_: 24.0 ± 0.6 mM; IC_50, rTRPV1_: ~ 12 mM). 2. Lidocaine not only activates both receptors, but also blocks both rodent TRPA1 and TRPV1 in a concentration-dependent manner. 3. Lidocaine seems to activate both TRPA1 and TRPV1 via an intracellular pathway. However, there also seem to be grave disparities between the mechanisms leading to activation of TRPA1 and TRPV1 by LAs. First, we found that repeated applications of 30 mM lidocaine induce an incomplete and partially calcium-dependent desensitization of TRPA1. In contrast, 30 mM lidocaine induced a strong but calcium-independent desensitization of TRPV1 [15]. The mechanism underlying these different patterns of lidocaine-induced desensitization of TRPV1 and TRPA1 is unclear. Second, we found that activation of TRPA1 by lidocaine was significantly abrogated in the triple mutant hTRPA1-3C. It is indeed established that reactive substances gate TRPA1 via a covalent and irreversible modification of these three intracellular cysteine residues [24,25]. Lidocaine, however, does not offer a molecular structure which would predict for such an effect. Furthermore, lidocaine-evoked TRPA1-currents display considerable faster current kinetics for both activation and inactivation when compared to TRPA1-currents evoked by the reactive agonists mustard oil and acrolein (Figure 1A). This fact could suggest that additional mechanisms, which are distinct from the covalent modification of cysteine residues, are crucially involved in LA-evoked activation of TRPA1. We found that LAs are also likely to interact with transmembrane region 5 (TM5) and that this interaction readily explains the species-specific differences between rodent and human TRPA1 with regard to current properties of lidocaine-evoked currents, i.e. rodent but not human TRPA1 display a resurging current following washout of the LA. While the same interaction was previously demonstrated for menthol and the general anesthetic propofol, only menthol seems to specifically interact with residues within TM5 to actually gate TRPA1 [23,31]. Similar to what we recently demonstrated for propofol [31], lidocaine still activates the menthol-insensitive mutant mTRPA1-S876V/T877L. Regarding TRPV1, we have convincing evidence that LAs activate TRPV1 by interacting with the intracellularly located vanilloid-binding domain and that this process requires an interaction of phosphatidylinositol 4,5-biphosphate (PI(4,5)P_2_) with the proximal C-terminal TRP domain of TRPV1 [15]. Neither of these two mechanisms can be applied on TRPA1 as it lacks both, a vanilloid-binding domain and a TRP domain within the proximal C-terminus [32]. However, TRPA1 can be directly activated by phospholipase C and also seems to be regulated by PI(4,5)P_2 _[33-35]. As judged from the existing literature however, the regulatory role of this signaling pathway seems to apply for most TRPA1-agonists and would thus not specifically apply for the effects evoked by LAs. As was previously reported by Karashima and colleagues [35], we also found that modulators of this pathway such as the PLC-blocker U73122 and the phosphatidylinositol 4-kinase blocker phenylarsine oxide directly gate TRPA1 and therefore impede a thorough characterization of these mechanisms on TRPA1 (data not shown, [35]). Nonetheless, PLC-PI(4,5)P_2_-signaling should be taken into regard in future studies which aim to identify common intracellular regulators of TRPA1 and TRPV1.

In a clinical perspective, this study complements a growing evidence that most general and local anesthetics as well as some analgesics activate or sensitize nociceptors via TRPA1 and/or TRPV1 [15,31,36-39]. Systemically applied substances, like general anesthetics and analgesics could employ TRPA1 to regulate or promote post-operative pain and inflammation. For example, a recent study suggested that TRPA1 plays an important role for airway inflammation and hyperreactivity in a mouse model for asthma [40]. Accordingly, TRPA1-activating volatile anesthetics were demonstrated to exert direct effects on bronchial dilatation in a TRPA1-dependent manner [39]. Moreover, a reactive metabolite of acetaminophen (N-acetyl-p-benzo-quinoneimine) activates TRPA1 and induces a TRPA1-dependent airway inflammation after systemic application of acetaminophen in a therapeutic dosage [41]. On the other hand, although local anesthetics are only locally applied, their concentrations should be sufficient to evoke a strong activation of both TRPA1 and TRPV1. When applied intrathecally, LAs can evoke a release of both glutamate and neuropeptides like CGRP from central nerve terminals expressing TRPA1 and/or TRPV1 and thus regulate spinal nociceptive signalling and ultimately promote "central sensitization" [11,15]. When applied intradermally or into the perineural space, LAs can promote the development of neurogenic inflammation by inducing a release of neuropeptides, thus sensitizing peripheral nociceptive signalling [12,15]. Furthermore, TRPA1 and TRPV1 display a significant co-expression in nociceptive sensory neurons [42]. It is therefore tempting to speculate about a high susceptibility of these neurons to undergo LA-evoked neurotoxicity as result of TRPV1 and TRPA1-activation. Although conclusive *in vivo *studies which address or confirm the proposed effects of general and local anesthetics are yet lacking, it is well conceivable that TRPV1 and TRPA1 antagonists will prove to be efficient for prevention and treatment of some unwanted effects which are likely to be induced by anesthetic substances that activate TRP-channels.

## Methods

### Mutagenesis and Heterologous Expression

Mutagenesis of human and mouse TRPA1 was performed as described previously [43]. Human TRPA1 cDNA was obtained from Dr. Paul Heppenstall (EMBL, Monterotondo, Italy), constructs of mouse and human TRPA1 were obtained from Dr. Ardem Patapoutian (The Scripps Research Institute, La Jolla, USA) and all other cDNAs were obtained from Dr. David Julius (UCSF, San Francisco, USA). All constructs were confirmed by DNA sequencing. HEK293t cells were transfected as described previously [43]. Briefly, HEK293t cells were transfected with plasmids of rat, mouse or human TRPA1 (5 μg) or TRPM8 (2 μg) along with a reporter plasmid (CD8-pih3m; 1 μg) by the calcium phosphate precipitation method. After incubation for 12-15 h, the cells were replated in 35 mm culture dishes and used for experiments within 2-3 days. Transfection-positive cells were identified by immunobeads (anti-CD8 Dynabeads; Dynal Biotech).

### Patch Clamp Recordings

Whole cell voltage clamp recordings currents were acquired with an Axopatch 200B amplifier (Axon Instruments/Molecular Devices, Sunnyvale, CA). Currents were filtered at 1 kHz and sampled at 5 kHz. Electrodes were pulled from borosilicate glass tubes (TW150F-3; World Precision Instruments, Berlin, Germany) and heat-polished to give a resistance of 1.5- 2.0 MΩ. The standard external solution contained (in mM): 140 NaCl, 5 KCl, 2 CaCl_2_, 2 MgCl_2_, 10 HEPES, 10 Glucose (pH 7.4 adjusted with tetramethylammonium hydroxide (TMA-OH)). In Ca^2+^-free solutions, CaCl_2 _was replaced by 5 mM EGTA. The internal solution contained (in mM) 140 KCl, 2 MgCl_2_, 5 EGTA and 10 HEPES (pH 7.4 adjusted with KOH). If not noted otherwise, cells were held at -60 mV. All recordings were performed at room temperature. Solutions were applied with a polytetrafluorethylen glass multiple-barrel perfusion system. The cold stimulus for experiments on TRPM8 was delivered using a multi-channel, gravity-driven system incorporating rapid-feedback temperature control. In this system, a platinum-covered glass capillary, positioned < 100 μm from the cell under study, was used as a common outlet [44]. The pCLAMP 8.1 software (Axon Instruments) was used for acquisition and off-line analysis.

### Chemicals

Lidocaine, procaine, mepivacaine (all Sigma-Aldrich) and QX-314 (Biotrend, Colonia, Germany) were directly dissolved in the extracellular solution. If not noted otherwise, the pH-values of all LA-solutions were corrected to pH 7.4 with TMA-OH. Capsaicin (Sigma-Aldrich) was dissolved in absolute ethanol to give a stock solution of 1 mM. Mustard oil, carvacrol (Sigma-Aldrich) and HC-030031 (Biotrend, Colonia, Germany) were dissolved in DMSO to give stock solutions of 100 mM.

### Statistical analysis

Calculations for statistical comparisons were performed with the Statistica software package 7.0 (Statsoft, Tulsa, USA) or with the Origin 7.0 software package (OriginLab Corporation, Northampton, USA). Tests used are stated in the text or in figure legends. *P *values < 0.05 were considered statistically significant. * denominates p < 0.05, ** denominates p < 0.01, *** denominates p < 0.001 and n.s. denominates a non-significant finding. All data are given as mean ± SEM.

## List of abbreviations

LA: local anesthetic; TRP: transient receptor potential; HEK293t cell: human embryonic kidney cell 293t; mTRPA1: mouse TRPA1; hTRPA1: human TRPA1; rTRPA1: rat TRPA1; TM5: transmembrane region 5; MO: mustard oil

## Competing interests

The authors declare that they have no competing interests.

## Authors' contributions

ALe planned and carried out experiments and drafted the manuscript. ALa, SK, and FN carried out experiments. CN coordinated the study, participated in planning the experiments, and drafted the manuscript. All authors read and approved the final manuscript.

## Supplementary Material

Additional file 1 **MO-induced currents of hTRPA1-WT are desensitized by lidocaine**. 10 mM Lidcoacine was co-applied with 100 μM MO during the steady-state phase of MO-evoked currents. Note that lidocaine first induced an additional activation followed by a desensitization. Cells were held at -60 mV. Click here for file

Additional file 2 **MO-induced currents of mTRPA1-S876V/T877L are not blocked by lidocaine**. 30 mM Lidcoacine was co-applied with 100 μM MO during the steady-state phase of MO-evoked currents and experiments were performed in Ca2+-free extracellular solution to minimize desensitization. Note the the resurging currents after washout of lidocaine. Cells were held at -60 mV. Click here for file

Additional file 3 **Lidocaine-induced currents of the calcium-insensitive mutant hTRPA1-L474A**. 10 mM (149 ± 23 pA, n = 7) or 30 mM (223 ± 67, n = 6) lidcoacine were applied for ~20s on separate cells to minimize desensitization. Note the resurging current after washout of 30 mM lidocaine. Cells were held at -60 mV. Click here for file
