# Supplementary figures and images for: Activation of TRPA1 by membrane permeable local anesthetics
Source: Mol Pain. 2011 Aug 23;7:62. doi: 10.1186/1744-8069-7-62 (PMC3179737; doi:10.1186/1744-8069-7-62)

## Slide 1
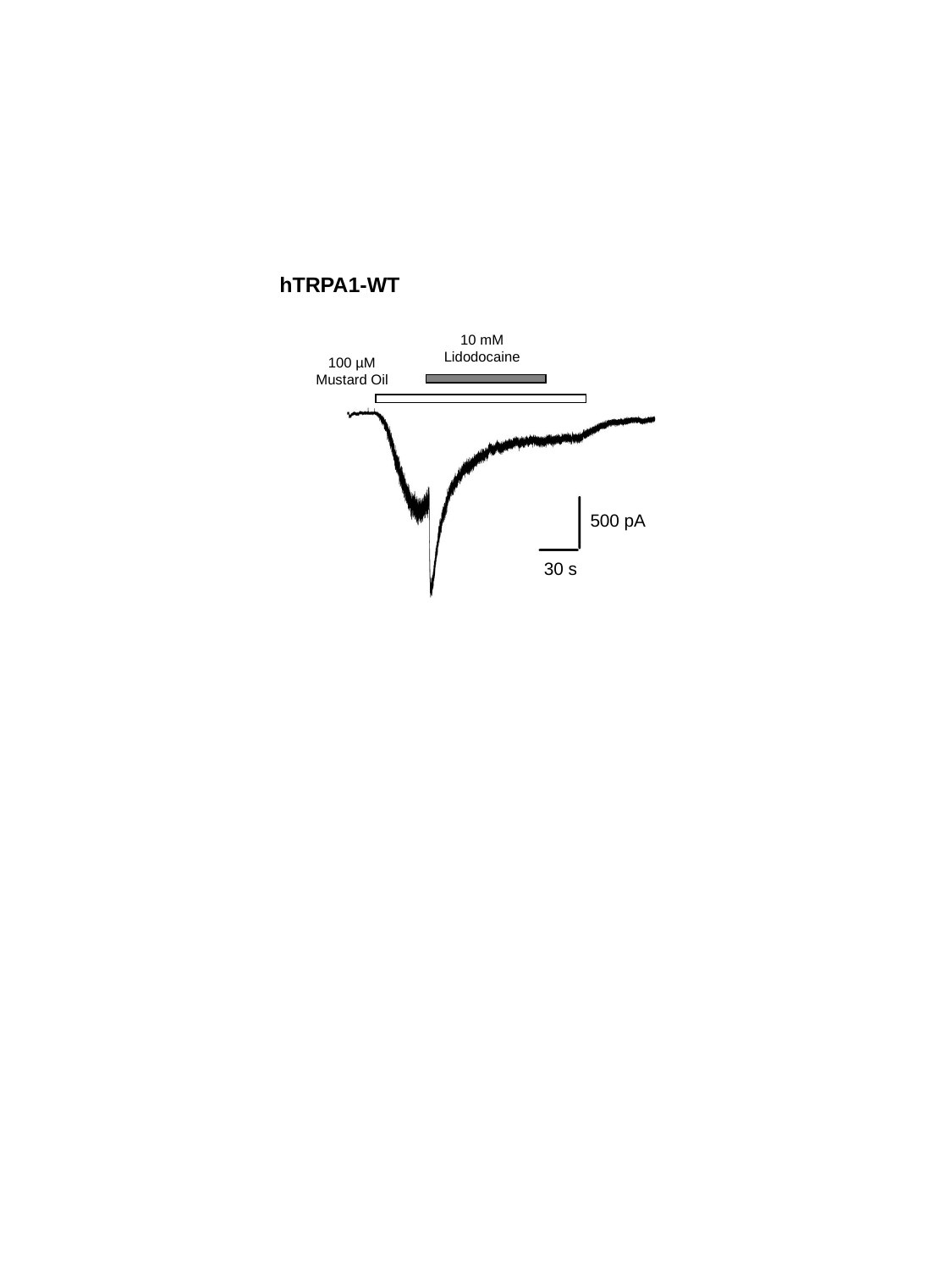

hTRPA1-WT
10 mM Lidodocaine
100 µM Mustard Oil
500 pA
30 s

Supplement: Additional file 1 — MO-induced currents of hTRPA1-WT are desensitized by lidocaine. 10 mM Lidcoacine was co-applied with 100 μM MO during the steady-state phase of MO-evoked currents. Note that lidocaine first induced an additional activation followed by a desensitization. Cells were held at -60 mV. [file 1744-8069-7-62-S1.PPT]

## Slide 1
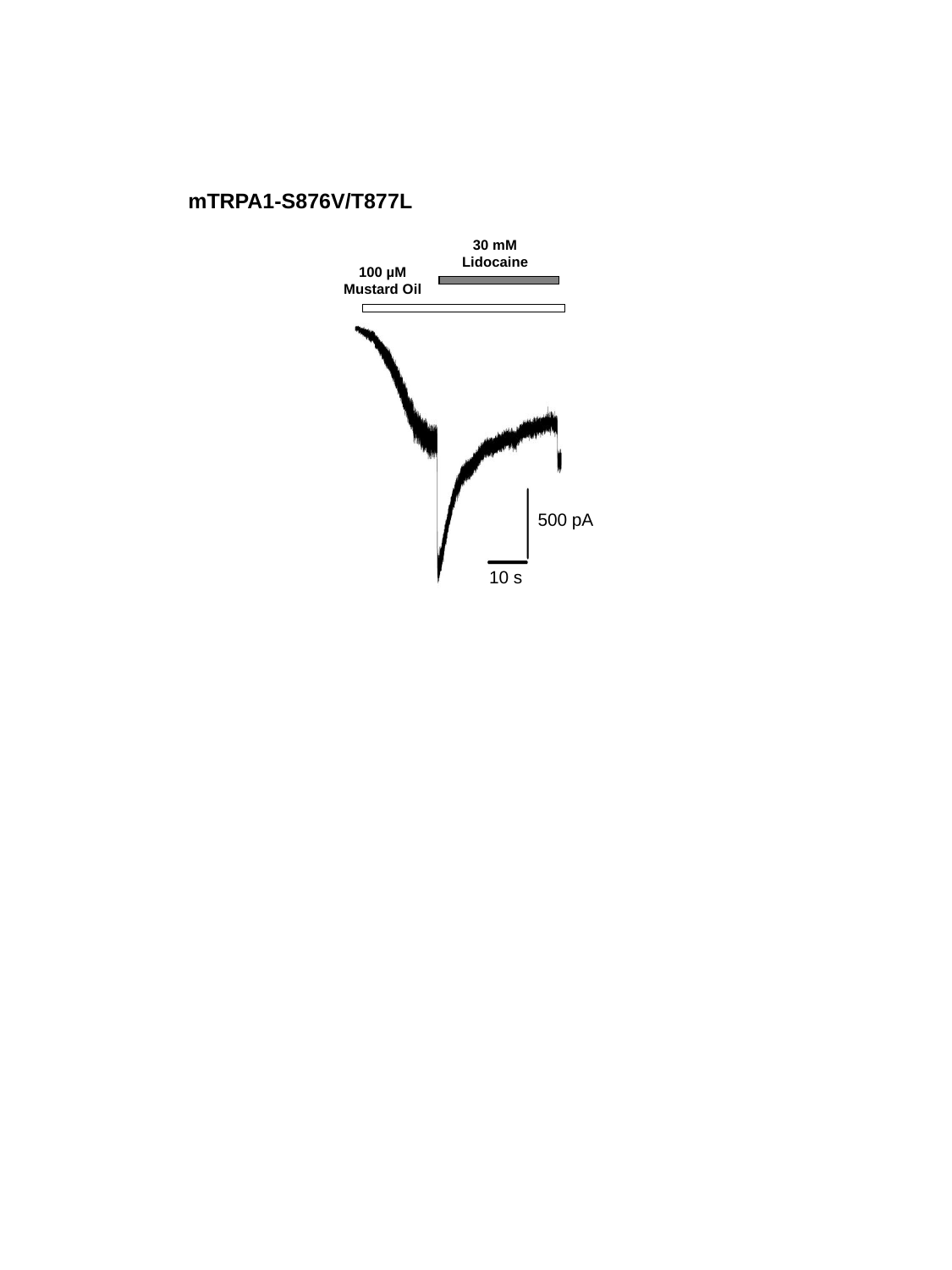

mTRPA1-S876V/T877L
30 mM Lidocaine
100 µM Mustard Oil
500 pA
10 s

Supplement: Additional file 2 — MO-induced currents of mTRPA1-S876V/T877L are not blocked by lidocaine. 30 mM Lidcoacine was co-applied with 100 μM MO during the steady-state phase of MO-evoked currents and experiments were performed in Ca2+-free extracellular solution to minimize desensitization. Note the the resurging currents after washout of lidocaine. Cells were held at -60 mV. [file 1744-8069-7-62-S2.PPT]

## Slide 1
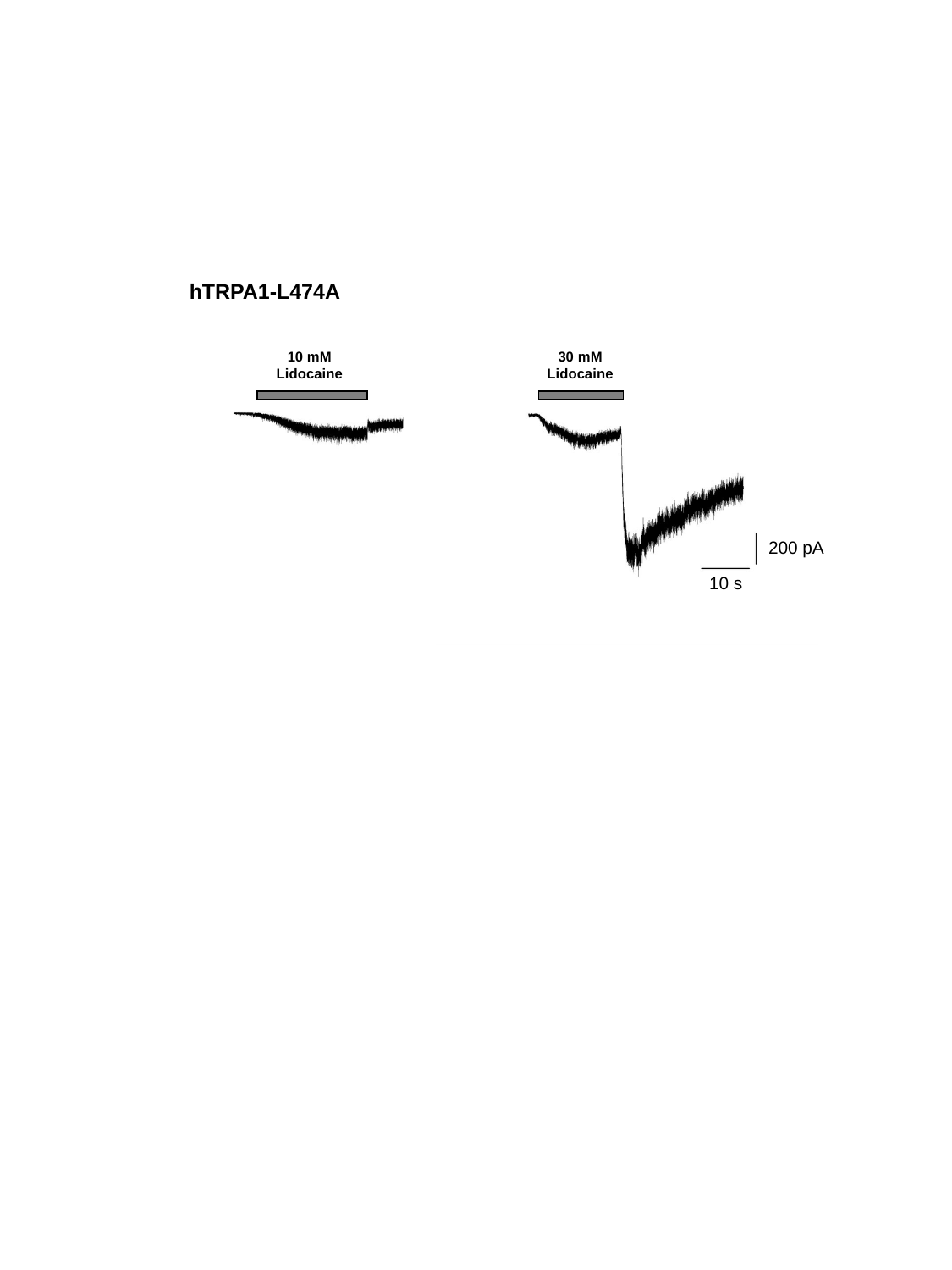

hTRPA1-L474A
10 mM Lidocaine
30 mM Lidocaine
200 pA
10 s

Supplement: Additional file 3 — Lidocaine-induced currents of the calcium-insensitive mutant hTRPA1-L474A. 10 mM (149 ± 23 pA, n = 7) or 30 mM (223 ± 67, n = 6) lidcoacine were applied for ~20s on separate cells to minimize desensitization. Note the resurging current after washout of 30 mM lidocaine. Cells were held at -60 mV. [file 1744-8069-7-62-S3.PPT]
